# Barriers and Levers to the Implementation of Napping During Night Shift Work in Healthcare Workers: A Qualitative Study in a French University Hospital

**DOI:** 10.1155/jonm/5279651

**Published:** 2025-12-09

**Authors:** Maryame Mazouz, Pauline Gouttefarde, Yanis Bouchou, Frédéric Roche, Carole Pelissier, Mathieu Berger

**Affiliations:** ^1^ Clinical and Exercise Physiology Department, University Hospital of Saint-Etienne, Saint-Etienne, France, chu-st-etienne.fr; ^2^ INSERM, IRD, SESSTIM, Aix-Marseille Université, Marseille, France, univ-amu.fr; ^3^ SAINBIOSE Laboratory (U1059 INSERM), Jean Monnet University, Saint-Etienne, France; ^4^ Chair in Elderly Health, PRESAGE Institute, Jean Monnet University, Saint-Etienne, France; ^5^ Gérontopôle Auvergne Rhône Alpes, Saint-Etienne, France; ^6^ UMRESTTE (UMR T9405), Lyon 1 University, Gustave Eiffel University, Jean Monnet University, Saint-Etienne, France; ^7^ Inter-Universitary Laboratory in Human Movement Biology (LIBM, EA 7424), Jean Monnet University, Saint-Etienne, France

**Keywords:** countermeasures, napping, night work, nurse, workload, workplace

## Abstract

**Introduction:**

Healthcare workers are exposed to health risks associated with shift work including cognitive impairments associated with decreased alertness, increased drowsiness, and disturbances in circadian rhythms. Although workplace napping has been shown to provide potential health benefits, its practical implementation within hospital settings remains limited. Thus, the aim of this study was to identify the barriers and levers to implementing a nap break at the workplace during night shift among nurses and care assistants.

**Methods:**

This qualitative study uses both individual and collective semistructured interviews and aims to identify perceived barriers and levers in implementing a dedicated space and break for napping during night work in a French University Hospital. The study assessed the feasibility of napping during night shift work for healthcare workers working 12‐h shifts and investigated perceptions of napping across different hierarchical levels, from management to prospective healthcare workers.

**Results:**

Twenty individual or collective interviews were conducted with 30 participants. Results showed that napping at work faced various barriers depending on the hierarchical level. Healthcare managers and the executive board were concerned about the challenges related to workload, lack of space, and insufficient staffing. Meanwhile, healthcare workers expressed cultural concerns regarding the negative perception of napping. Our findings revealed differences in napping perception between managers and administration, as well as a lack of exchange between hierarchical strata.

**Conclusion:**

This study suggests that a shift in the perception of napping and improved communication within the hospital could facilitate its integration during night work. Collaboration among all hierarchical levels and a strong commitment from leaders are needed to overcome these challenges.

## 1. Introduction

Longer working hours have led to increased health risks, with the International Labor Organization (ILO) and the World Health Organization (WHO) reporting 745,000 deaths linked to this phenomenon in 2016. This global health problem highlights the need to re‐evaluate work schedules, particularly for night workers [1]. In France, approximately 3.5 million people, accounting for up to 25% of the active workforce, are engaged in night shift work, of which 42% are healthcare workers [2]. Shift work is common among healthcare professionals to ensure continuity of care and requires a rotation among teams with the same position to maintain continuous services [3].

Current research indicates that night work can impair health and reduce quality of life [4]. The sleep deprivation associated with shift work leads to cognitive and sleep disorders, decreased alertness, increased drowsiness, and the risk of chronic diseases [5–7]. Thus, recent studies highlight the urgent need for action to mitigate nurse fatigue and minimize risks for patients.

Napping, which could be used as a countermeasure, has proven beneficial in reducing sleep deprivation and maintaining the cognitive performance of healthcare providers [8, 9]. Although highly favored by a majority of healthcare workers with over 90% approval, its implementation remains unusual in French and Canadian hospitals, often hampered by biases related to the demands of patient care and safety, staffing needs, and organizational and environmental factors [10]. Research in Canada highlighted the challenges of integrating napping into hospital routines, particularly in terms of patient safety and care organization [11]. These difficulties reflect the specificity of barriers to its adoption in healthcare settings [12]. However, the adoption of napping could enhance attractiveness of hospitals and strengthen the retention of nursing staff, aligning with the ‘magnet hospital’ concept that retains and attracts professionals [13].

Although napping within hospitals is recognized as beneficial for healthcare workers’ health and recommended by health authorities, its adoption remains limited [14], revealing a gap between the recognition of its health benefits and its practical implementation. Similarly, even if the French Agency for Food, Environmental, and Occupational Health and Safety and the High Authority of Health support its practice to combat drowsiness, especially among night shift workers, napping has not been yet officially integrated into hospital night routines, highlighting the need for further research to clarify the modalities of its implementation [3, 14, 15].

This is compounded by a lack of knowledge about the barriers and levers to its implementation, particularly across different hierarchical levels (from nurses to managers, from directors to nursing students and future managers). To our knowledge, few studies have explored these hierarchical dimensions in depth to understand the specific dynamics that facilitate or hinder the institutionalization of napping at work.

Thus, the purpose of the present study was to identify the barriers and levers to implementing a napping time at the workplace during night shift for hospital staff (nurses and nursing assistants) by interviewing different hierarchical levels involved in a French University Hospital.

## 2. Methods

### 2.1. Study Settings

This study was based on a qualitative approach, using semistructured interviews, either individual (30 min) or group‐based (1 h) [16]. The qualitative method was chosen to enable a deep and nuanced exploration of participants’ experiences and perceptions, essential for capturing the complexity of the phenomena under study. The study was performed in one of the main university hospital in the Auvergne Rhône‐Alpes region in France.

### 2.2. Sampling

The study used purposive sampling, including nurses and healthcare managers working 12‐h shifts, as well as members of management responsible for managing healthcare resources and policies. This method was chosen for its strategic fit with the study objectives. Final‐year students from the “Institute for Health Management” Training ready to take on management roles in hospitals, and third‐year nursing school students, close to entering professional life, were also interviewed in order to gather their perceptions.

Initial contact with the participants was held either by advertisement or by email, and information sessions were conducted to introduce the study’s objectives, to obtain participant consent, and to schedule interviews.

### 2.3. Interview Guide

The guide aimed to explore participants’ views and practices regarding napping at work, as well as potential barriers, focusing on current behaviors and identifying levers for integration in the workplace. A specific guide was created for each hierarchical level surveyed. Before its implementation, the interview guide was tested about 10 times with nurses, physicians, and managers to ensure its clarity and relevance. Following this preliminary test, it was adjusted to better meet the study’s objectives. Once the official interviews began, the guide underwent no further modifications.

Interviews were recorded to ensure accurate transcription. As part of the interview guide, a specific segment was dedicated to discuss the setup of an “ideal” napping room. Participants were asked to evaluate and rank a variety of equipment and ambiance options using photos (see Figures A1 and A2 in appendix for further details).

### 2.4. Data Collection and Recruitment

Prior to each session, oral informed consent was obtained, highlighting the voluntary nature of participation and the lack of consequences for refusal. The interviews were recorded and destroyed after transcription by the principal researcher. Codes were assigned to participants to ensure their anonymity and protect their privacy throughout the study.

### 2.5. Ethical Considerations

This qualitative study received ethical approval from the local ethics committee of the University Hospital of Saint‐Etienne (IRBN 232023/CHUSTE) prior to data collection from May to July 2023. All participants received clear and understandable information about the objectives and the course of the study from the investigator and via an information letter. They were free to accept or refuse to participate in the study.

### 2.6. Data Analysis

Thematic analysis was applied as the primary methodology for data exploration. The software NVivo 14 was used to structure and facilitate the analysis. Data collection continued until data saturation was reached, meaning that no new themes emerged from additional interviews. After meticulous transcription of the interviews, they were shared with participants for validation, as recommended in COREQ Item 23, and re‐read in parallel with the audio recordings to ensure transcription fidelity by the research team [17]. Only data relevant to the study’s objective were selected. At this step, the COREQ checklist was followed to ensure rigorous collection, analysis, and reporting of qualitative data.

### 2.7. Reliability

The investigators meticulously transcribed and checked each transcription, analyzing the transcripts to identify preliminary themes using a thematic analysis approach. This initial step was followed by consultation with five other researchers, experts in different fields (medicine, sociology, psychology, sports and sleep research, and public health), to discuss each theme. In‐depth discussions were conducted to confront and harmonize these themes, resolving any divergences until a consensus was reached. The research team worked together to minimize potential biases in the analysis and interpretation of data [18].

Finally, a part of the transcription was shared with some participants (including members of the management) to verify, clarify, or obtain additional information, thus ensuring that the transcription authentically reflected their opinions and experiences.

## 3. Results

The demographic characteristics of the participants are presented in Table 1. Of the 30 participants included in this study (mean age ± SD, 42 ± 10.5 years), a majority were female (74%), and the average tenure was 11.1 ± 0.1 years. The distribution of professions was as follows: 7 nurses, 5 health managers, 5 members of the management team (including 3 directors and 2 seniors health managers), 9 trainees at the Institute for Health Management Studies, and 4 trainees at the Institute for Nursing Care Studies (Table 1).

**Table 1:** Participant characteristics.

	Health manager (*n *= 5)	Nurses (*n *= 7)	Executive staff (*n *= 5)	Health management student (*n *= 9)	Nursing student (*n *= 4)	Total (*n *= 30)
Female (%)	5 (100%)	5 (60%)	3 (60%)	6 (67%)	4 (100%)	23 (74%)
Age (years)	46.6 ± 7.4	40.0 ± 10.2	52.2 ± 5.3	46.6 ± 7.4	32.6 ± 10.5	42.1 ± 10.5
Tenure in role (years)	9.8 ± 8.0	11.6 ± 7.1	11.8 ± 5.2	N/A	N/A	11.1 ± 0.9

*Note:* Data are presented as mean ± standard deviation or *n* (%).

Overall, five themes and 11 subthemes were identified and are described in Table 2.

**Table 2:** Themes and subthemes derived from interviews with healthcare workers on night shift napping.

Themes	Subthemes
Theme 1: Understanding the practices and strategies to combat drowsiness	Manifestations and management of drowsiness by healthcare staff
	Unregulated practice of napping and its conditions

Theme 2: Challenges of implementing a nap room in a hospital setting	Professional constraints as potential barriers to napping
	Logistical constraints and financial and staffing challenges
	Ensuring continuity of patient care through optimized organization
	Engaging key stakeholders from the start

Theme 3: Legal, ethical, and equity challenges	Legal concerns
	Equity within the workforce
	Promoting napping: Boosting well‐being and staff loyalty in hospitals

Theme 4: Perceptions and acceptability of napping at work	Benefits of napping
	Consensus and resistance to napping
	Terminology and public perception

Theme 5: An optimized space for napping	Nap cocoon preference
	Improved comfort
	Management and hygiene concerns

### 3.1. Theme 1: Understanding Practices and Strategies to Fight Sleepiness

#### 3.1.1. Manifestations and Management of Drowsiness by Healthcare Staff

All the nurses expressed experiencing chronic fatigue during their night shifts, which also affects their personal lives, even on their days off. Health managers, reflecting on their past experience as nurses, also confirmed this fatigue, a topic frequently discussed during annual interviews. However, only a minority of the management team acknowledges this issue.

*“From around 3 a.m., it’s as if you feel like a kind of bradycardia where everything slows down: movements, thoughts, the heart. You go into hibernation mode.”* (Healthcare managers, Female)

#### 3.1.2. Unregulated Practice of Napping and Its Conditions

To counteract fatigue and sleepiness, they adopt practices such as informal, spontaneous naps in precarious conditions (on tables, in patient chairs, or even on the floor) and consume large amounts of caffeine.

*“These are micro-naps; you don′t realize you are fallen asleep until you suddenly wake up thinking, oh no! (…) You′re talking, and then you see a colleague putting his head between his arms. You know he’s going to fall asleep, and then he’s gone for 10 minutes.”* (Nurse, male)

These observations were reported by all hierarchical levels involved in the study, except for some directors who categorically assert that healthcare workers do not engage in informal napping.

*“It′s normal that I am not aware [of the napping practice at work].”* (Management team member, male)

### 3.2. Theme 2: Challenges of Implementing a Nap Room in a Hospital Setting

#### 3.2.1. Professional Constraints as Potential Barriers to Napping

Numerous concerns specifically related to the practice of napping were expressed, including the fear of sleep inertia, difficulties in falling asleep, and managing nap time. Several participants reported concerns regarding ‘professional awareness’ of not leaving their patients unattended. These concerns were shared by different participant groups, with the exception of some members of the management.

*“′I′m afraid to fall asleep and then being completely out of sync. If a patient has a cardiac arrest, and I′m abruptly woken up to handle that situation, it′s tricky.”* (Nurse, male).

For most nurses and a significant number of health managers, workload fluctuations, especially in intensive care units which promotes constant stress levels, make the use of a nap room complicated.

*“You never really disconnect, even at rest, you′re constantly monitoring patients. You are under constant stress as soon as you enter ICU.”* (Nurse, male)

#### 3.2.2. Logistical Constraints and Financial and Staffing Challenges

A point raised specifically by participant groups involved in management, such as health managers and directors, was the lack of available space within the services to set up nap rooms.

*“Here, at the hospital, every square meter is expensive. We′re continuously looking for space for offices, for new services.”* (Members of the management team, male)

Additionally, the limited staffing of healthcare workers is an additional barrier to allocating time for napping.

*“For me, it would require adding a caregiver. I don′t see how, with a constant workforce, we can integrate it.”* (Healthcare manager, female)

Some directors indicated that increasing staffing was impractical due to high costs, particularly if this measure was generalized to all university hospital services. They also added that this would not compensate for absenteeism within the hospital.

*“Several tens of millions of euros for all services would be necessary if we wanted to generalize the addition of a position that would entail 6 full-time equivalents extra per shift and per service.”* (Member of the management team, female)

#### 3.2.3. Ensuring Continuity of Patient Care Through Optimized Organization

A consensus emerged among all supervisors, except for some nursing managers: The possibility of optimally organizing the caregiving team to allow a caregiver to take a nap during the quieter periods of the night shift (between 2 and 5 a.m.), while ensuring continuity of patient care.

*“Yes, I think it′s more feasible towards the end of the night shift [to take a nap]. If we split the patients like we already do during lunch time…” »* (Nurse, female)

#### 3.2.4. Engaging Key Stakeholders From the Start

To successfully implement this project, some members of the management team recommended close collaboration with the university hospital management from the early stages of its development to foster the adherence of all involved stakeholders.

*“It′s necessary that the Care Department and the Human Resources Department are involved from the beginning of this discussion. Not just the health managers of units, who might be favorable, but also the senior health managers and the human resources and nursing management.”* (Member of the management team, male)

### 3.3. Theme 3: Legal, Ethical, and Equity Challenges

#### 3.3.1. Legal Concerns

Some health managers and future managers expressed concerns about potential legal risks of introducing nap breaks at work, which could lead to a perception of ‘understaffing’ and potentially result in legal proceedings from patients’ families. This view was not shared by the management, except for one director who fears media repercussions:

“*If I was a patient′s family, if I knew about such an arrangement and could prove that the service was understaffed and there was an incident, the hospital and everyone would be in trouble.”* (Health Management trainee, female) and “*Let′s say there′s a death, if the family learns that the team was sleeping at that time, even if it has nothing to do with the patient′s death, it could be complicated to handle from a media standpoint.”* (Member of the management team, female)

#### 3.3.2. Fairness Within the Workforce

Managers and directors expressed various concerns. A primary fear was that creating nap rooms could lead to inequalities between services and exacerbate the difficulty in recruiting staff if the rules were not extended to all services:

*“If you don′t apply the same rules [in all services], it could create a feeling of inequality.”* (Member of the management team, female)

#### 3.3.3. Promoting Napping: Boosting Well‐Being and Staff Loyalty in Hospitals

However, others believe that promoting napping in hospitals could improve workplace well‐being, alleviate the constraints of intense work rhythms, and reduce turnover:

*“Yes, I think [the management should encourage napping], even though it seems unbelievable to me that this would happen. But if it′s for the well-being of your teams, and it improves things and stops people from leaving their position because of that [the work rhythm], then yes.”* (Nurse, female)

### 3.4. Theme 4: Perceptions and Acceptability of Napping at Work

#### 3.4.1. Benefits of Napping

Healthcare providers and health managers, as well as nursing students and some members of the management, recognized the potential benefits of napping for concentration, quality of care, team cohesion, and reducing the risk of road accidents. For some members of the management, there was no evidence that healthcare workers were making more mistakes at night than during the day, and therefore, they expressed the need of evidence‐based studies:

*“The agent would be more alert, with an improved level of vigilance (…)”* (Healthcare manager, female)

*“When we observe adverse events, we see that there are many more during the day than at night. That doesn′t necessarily mean that all adverse events are solely due to a decrease in vigilance.”* (Member of the management team, male)

*“If we have convincing studies (…) it could serve as an anchor point (…)”* (Healthcare manager, female)

#### 3.4.2. Consensus and Resistance to Napping

Within the health care environment, a consensus has emerged among nurses and health managers that napping is generally viewed positively by healthcare managers.

*“No, I don′t think it′s frowned upon [by the healthcare workers]”* (Healthcare manager, female)

Despite this positive perception, resistance from some quarters persists. A number of team leaders have pointed out that their team’s environment does not support rest or napping, often due to inadequate facilities such as inappropriate chairs.

“[Deckchairs] have always been refused by the chief” (Healthcare manager, male); *“It′s true that it remains something frowned upon. You′re at work, you′re there to work.”* (Healthcare manager, female)

However, amidst these challenges, there is a glimmer of flexibility. Some healthcare managers exhibit a partial tolerance for napping:

*“It′s true that we rest, but yes, apparently, it can be tolerated by the management.”* (Nurse, female)

#### 3.4.3. Terminology and Public Perception

Participant groups (excluding healthcare providers) questioned the semantics of the word ‘nap’ due to its possible pejorative connotation with the public. They suggested that using another term, such as ‘rest time’ or ‘recovery time,’ might be more appropriate:

*“The term ‘nap’ is not necessarily appropriate. However, recovery time, yes (…) It will be handled differently, both by the staff and by the management”* (Health Management trainee, female)

### 3.5. Theme 5: An Optimized Space for Napping

#### 3.5.1. Nap Cocoon Preference

Most participants, across all hierarchical levels, consider the ‘ideal’ nap room to be a quiet, dark, and private space near their services. The majority advocated for the use of nap cocoons to meet the aforementioned needs, with the added benefit of physical rest since the feet were elevated, promoting venous return:

*“The nap cocoon, I′d say this one because it seems to allow a fairly extended position. You can put the blanket on to be quieter and in your own little space with the little table to put things on, that’s nice.”* (Nurse, female)

Members of the management team agreed that comfort was crucial for the adoption of such a space:

*“The most important thing is comfort, from the professional′s point of view, because if somebody has a rest area that doesn’t suit him, it won’t be used.”* (Member of the management team, male)

#### 3.5.2. Improved Comfort

To improve comfort during napping, some healthcare workers and health managers suggested to have at their disposal blankets, privacy screens, blackout masks, and an environment that promotes relaxation with a “zen” decoration:

*“(…) Make it possible to cover up”* and *“Folding screens and a little peace and quiet for everyone”* (Healthcare manager, female) and *“I can′t sleep if there′s light. However, I see (on the photo) she has something over her eyes, so that could also be a solution.”* (Healthcare manager, female)

#### 3.5.3. Management and Hygiene Concerns

However, some health managers have expressed concerns about the hygiene of the nap room, but others proposed a solution involving the use of sanitizer:

“Who’s going to manage the room? It would need an external person to manage it (*…*) (Nurse, female)

## 4. Discussion

This study, conducted with individual and collective interviews within French hospital staff (healthcare workers, directors, and senior and junior health managers), aimed to identify the barriers and levers to establishing a nap room and nap time during night shifts. Among the barriers to nap implementation, a usually negative perception of napping and a mismatch with French culture were raised, as well as financial challenges for additional recruitment necessary for the continuity of care. The adoption of napping in hospitals is also hampered by organizational challenges, such as the fear of insufficient staffing during napping periods, a problem already mentioned by previous studies in Canada [11, 14]. Logistical constraints, such as lack of space, were also mentioned.

However, the majority of participants included in the present study acknowledged the benefits of napping in enhancing alertness and care quality [19]. Of note, although napping is not a formalized practice, it is already a spontaneous practice among healthcare workers.

To promote its implementation during night shift work, a reorganization of human resources was suggested. Regarding the location of the nap room, the majority preferred a location outside the service, conceived as isolated and comfortable, equipped for optimal rest while respecting hygiene standards.

Our results revealed that more than half of the health managers were aware of the risks associated with fatigue for the safety of both patients and healthcare workers themselves [20]. The difficulty for healthcare workers to step away from their patients for a break, exacerbated by downsizing, particularly at night, was often linked to the facility’s culture and poor perception of it [21]. A reorganization of teams, including rotations or shared patient care, was proposed by participants as a solution. This strategy, also suggested in the industrial sector by Muzet et al. [22], would allow for sleeping time while others maintain surveillance.

Our results also highlighted that the lack of dedicated space for napping was a major barrier to the establishment of nap rooms. Other studies also underlined the need to rethink hospital structure to incorporate appropriate rest spaces [14]. Nurses emphasized that nap rooms should be located near clinical units to allow quick response in case of emergencies, while also being located in environments conducive to restorative rest. Implementing mini‐training sessions to standardize napping, combined with sleep hygiene practices and relaxation techniques, could improve its effectiveness [21]. Additionally, Watanabe et al. reported the lack of adequate conditions for napping, such as sleeping on a chair, an obstacle also mentioned by our study’s participants [23].

As already demonstrated by Muzet et al. in the industry sector [22], our study confirms that the negative perception of napping in France, often associated with a lack of professional ethics and laziness, is a major barrier to its implementation in the healthcare sector. Healthcare managers seem to be more open to this practice, unlike the executive board who are often opposed, as mentioned by Edwards et al. [11]. Our results revealed that most healthcare managers are aware of the regular but unofficial practice of napping among their teams. However, some members of the management admitted that they had not been informed about the practice of napping within their department. This discrepancy reflected a lack of communication between hierarchical strata, as also highlighted in a study concerning the communication between future health managers and management [11]. Establishing a cross‐strata steering committee could facilitate the identification and application of appropriate solutions [24].

Cultural perceptions of napping varied significantly from country to country. In Japan, napping is common and valued as a sign of diligent work, and napping is even tolerated during business meetings [20]. Conversely, in Canada and in the United States, napping is often seen as a waste of time, as it can be in France [11, 12]. To facilitate its implementation at work, some participants suggested to rename napping into ‘rest period’ or ‘recovery time.’ This terminological change could help to improve the negative perception of this practice and promote its integration into the professional environment. This proposition seems suitable to redefine a beneficial practice from a more acceptable perspective. This new lexical approach could help lift some institutional and cultural barriers.

Implementing napping during regulated breaks for healthcare workers is crucial, particularly for mitigating risks associated with night shift work. In France, labor laws mandate that healthcare providers are entitled to 20‐min breaks every 6 h, thereby providing a legal framework for structured naps^1^. Some studies, in particular those by Ruggiero and Redeker and Barthe et al., showed that napping is already a common practice, especially between 2 and 4 a.m., a period of decreased activity [7, 25]. Thus, aligning naps with these legal breaks could allow to optimize healthcare workers rest while respecting legal provisions.

## 5. Study Limitations and Future Perspectives

This study has some limitations. First, the sampling was restricted to healthcare workers of one university hospital and may not represent the position in another hospital or professional sector. Moreover, perception of napping varies from culture to culture and the barriers and levers identified in the present study in France may not be applied in another country. Secondly, there may have been a selection bias since participation was voluntary. The self‐reported data process may have led to a possible social desirability bias. Although, interviews were anonymized and performed by an independent researcher, some participants may not have felt free to give their own position on napping in the workplace. Nevertheless, the present survey provided insights into napping in a hospital setting and can guide future interventions/solutions to promote its implementation in healthcare services.

## 6. Conclusion

Our study showed that the perception of napping may differ from one hierarchical level to the other. Although almost everyone agrees regarding the benefits of napping, cultural, logistical, and institutional barriers remain to be overcome. In particular, healthcare workers highlighted the need of dedicated spaces for napping and rotating schedule to ensure continuity of care, but lack of facilities is a major problem at the hospital. In future, the creation of suitable rest areas should be considered when creating or renovating hospital departments. Our study further emphasizes the pivotal role of management and hospital organizations in actively supporting the implementation of such practices. Further interventional studies are needed to legitimize and frame the possibility to nap at work.

In conclusion, to establish the optimal conditions for napping during night shift breaks, it is essential to adopt a multilevel approach, supported by steering committees that address organizational, cultural, and environmental dimensions.

These steps could significantly improve the well‐being and efficiency of healthcare workers, thus addressing major challenges in the current healthcare sector.

## Conflicts of Interest

The authors declare no conflicts of interest.

## Funding

The research did not receive funding from any sources.

## Endnotes

^1^Article L4121‐2—Code du travail—Légifrance (Internet) (cited 2023 July 25). It is available at the following link: https://www.legifrance.gouv.fr/codes/article_lc/LEGIARTI000033019913/2022-07-08).

## Supporting Information

Figure A1: Nap equipment proposed to participants.

Figure A2: Nap room environment proposed to participants.

These figures illustrate the different types of nap equipment and environments presented to participants during the interviews to help them visualize and discuss optimal napping conditions in hospital settings.

## Supporting information


**Supporting Information** Additional supporting information can be found online in the Supporting Information section.

## Data Availability

The data that support the findings of this study are available upon reasonable request from the corresponding author (MB).
