# Supplementary material for: Barriers and Levers to the Implementation of Napping During Night Shift Work in Healthcare Workers: A Qualitative Study in a French University Hospital
Source: J Nurs Manag. 2025 Dec 9;2025:5279651. doi: 10.1155/jonm/5279651 (PMC12714173; doi:10.1155/jonm/5279651)
Supplement: Supplementary file 1 — Supporting Information Additional supporting information can be found online in the Supporting Information section. [file JONM-2025-5279651-s001.docx]

**SUPPLEMENTAL MATERIAL**

**Barriers and levers to the implementation of napping during night shift work in healthcare workers: A qualitative study in a French University Hospital**

Maryame Mazouz, Pauline Gouttefarde, Yanis Bouchou, Frédéric Roche, Carole Pélissier, Mathieu Berger

**Online Data supplements**

- 2 Supplemental Figures

**Figure A1.** Nap equipment proposed to participants

**Figure A2.** Nap Room environment proposed to participants
